# “If money was no object”: A qualitative study of South African university office workers’ perceptions of using height-adjustable sit-stand desks

**DOI:** 10.17159/2078-516X/2022/v34i1a13881

**Published:** 2022-01-01

**Authors:** PJ Gradidge, M Phaswana, JY Chau

**Affiliations:** 1 Centre for Exercise Science and Sports Medicine, Faculty of Health Sciences, University of the Witwatersrand, Johannesburg, South Africa; 2 Department of Health Sciences, Faculty of Medicine, Health & Human Sciences, Macquarie University, Sydney, Australia

**Keywords:** sit-stand desks, university office-based workers, workplace, sedentary behaviour, South Africa

## Abstract

**Background:**

Data from empirical investigations on the feasibility and acceptability of using sit-stand desks in an office-based setting in low- and middle-income settings are limited.

**Objectives:**

To explore the perceptions of South African office workers towards using height-adjustable sit-stand desks to reduce sitting time during vocational hours.

**Methods:**

Self-reported sedentary behaviour and in-depth, semi-structured interviews were conducted in December 2020. Thematic content analysis approach was used to develop themes.

**Results:**

Eleven office workers with a work-time sitting time of 8 (6–8) hours were interviewed (age 40.5 ± 12.6 years), most (91%) were female. The main themes emerged and included: overall impressions of the height-adjustable sit-stand desks; enablers versus barriers to using the desk and readiness to continue using sit-stand desks.

**Conclusion:**

The findings of this research add to the evidence on environmental workstation modifications for reducing sedentary behaviour. Further investigations on the efficacy of sit-stand desks are recommended in South African university office workers.

Sedentary behaviour is a growing global public health concern. Elevated levels of sitting time are associated with all-cause mortality and cardiovascular disease risk factors, especially among people who are not sufficiently active. ^[1]^ Sedentary behaviour is defined as sitting or lying recumbent or such activities that result in energy expenditures of ≤ 1.5 metabolic equivalents. ^[2]^ Obesity and related comorbidities have strong links with sedentary behaviour, particularly in low-income and middle-income countries (LMICs), such as South Africa, where populations continue to shift into obesogenic urban environments and adopt these sedentary lifestyles. ^[3]^ Recent data demonstrate that the prevalence of South Africans sitting ≥ eight hours per day is approximately 4.6% of the population, and this is mostly among those living in urban areas. ^[4]^ Office workers in South Africa are prone to sitting for long periods of time during vocational hours, ^[5]^ that are similar to high-income countries (HICs) where employees are sedentary for at least two-thirds of the workday. ^[6,7]^

A recent systematic review using pooled data reported that interventions for reducing sitting in office workers have found small improvements in cardiovascular health, particularly with systolic blood pressure (−1.1 mm Hg), body composition (body weight: −0.6 kg; body fat percentage: −0.3%; waist circumference: −0.7 cm, and lipid profile (high-density lipoprotein cholesterol: 0.04 mM) and insulin (−1.4 pM). ^[8]^

Interventions in free-living environments, including workplaces, that target sedentary behaviour alone or in conjunction with physical activity, are effective for improving biomarkers associated with cardiometabolic risk profiles. ^[8]^ Most of the sedentary behaviour interventions have been carried out in high-income countries and Eurocentric populations. The evidence indicates that sit-stand workstations are effective in workplace strategies in high-income country settings. ^[8]^ Little is known about the feasibility of this strategy in the context of low-middle income countries, possibly due to the comparatively longer duration of interventions conducted in the studies in high-income countries. ^[8]^ For example, an Australian workplace intervention that included environmental modifications (sit-stand desks), messaging to encourage behaviour adjustment and health coaching observed significant reductions in occupation-related sitting time and cardiometabolic biomarkers at 3- and 12-months. ^[9]^

South African workers (n=1954) recruited from 18 companies were estimated to have a high prevalence of non-communicable diseases due to the growing obesity epidemic in the country. ^[10]^ Hene et al. also reported that 67% of workers in their study were overweight, while 77% were insufficiently physically active. ^[11]^ What is lacking, however, is the comprehension of how tools to disrupt occupation-related sitting are feasible for workers in South Africa. To our knowledge, strategies using sit-stand desks have not been applied in the South African workplace and not in a university context in particular. Therefore, we sought to target this knowledge gap by exploring the perceptions of South African university office workers regarding the feasibility of sit-stand desks to reduce sedentary behaviour at work.

## Methods

### Setting, design, participants and recruitment

This study was conducted at the Faculty of Health Sciences, University of the Witwatersrand, Johannesburg, South Africa. The study aimed to assess the feasibility of an environmental modification to promote less sitting using sit-stand desks.

On the 20^th^ of November 2020, all office staff from one building in the Faculty of Health Sciences were invited by email to participate in this study. Of the thirty-two potential participants who were working in the office during the COVID-19 lockdown, 11 responded to the invitation and completed an online pre-screening survey. The email invitation included a participant information sheet and a consent form. Ethical approval was obtained from the University of the Witwatersrand (ethics certificate number M190224). Written consent was provided by all participants.

The inclusion criteria included adults (aged above 18 years) with access to a desk or workstation within an office, the ability to communicate in English, the ability to walk or stand for at least 10 minutes, and individuals who worked in the office for at least three days a week.

The sit-stand workstation consisted of a height-adjustable workstation (JUMBO DeskStand™, DeskStand, South Africa) that allowed office workers to vary their posture throughout the workday between sitting and standing for a period of two weeks. The participant’s workstation was set up by one of the investigators (MP) in the best ergonomic position in relation to the participant’s height and needs. Upon installation, participants were educated on the benefits of standing-based work and interrupting sitting time. Participants were provided with training on how to optimally set up the workstation for their own individual job roles. The participants were asked to disintegrate their sitting time by accumulating bouts of standing activities of at least 10 minutes initially and, then progressing to longer bouts of 30 minutes or more as the study progressed.

### Data collection procedure

Participants were first asked to self-report their estimated time spent sitting (hours) in various aspects of sitting during work, commuting and at home using an adapted version of the Workforce Sitting Questionnaire (WSQ). ^[12]^ The WSQ is reliable and has been validated for use in office-based workers. ^[12]^

The semi-structured interviews took 10–30 min each, and were all recorded and conducted in English. Interviews were conducted by one researcher (MP) using a semi-structured interview guide (Supplementary File 1) via Microsoft teams or in-person between 1^st^ of December 2020 to 19^th^ February 2021 as preferred by the participants during the Covid-19 pandemic. Recorded audio files from the discussions were transcribed verbatim. All transcripts were checked against the recordings to verify accuracy and credibility, and grammatical editing was adopted where necessary.

### Data credibility and trustworthiness

The authors followed and adopted the Eight “Big-Tent” criteria for excellent qualitative research in conducting this study. These criteria included a worthy topic, rich rigour, sincerity, credibility, resonance, significant contribution, ethical and meaningful coherence. ^[13]^ Exploring the perceptions of using height-adjustable sit-stand desks was considered *a worthy topic* to inform environmental sedentary behaviour interventions in the South African context. Regarding *rich rigour*, the authors followed the established methodology for data collection, processing, and analysis. *Sincerity* was observed by the authors that confirmed that the interviews were transcribed correctly and processed using recognised software (Atlast.ti) and that there was agreement on the themes and sub-themes to ensure optimal trustworthiness. *Credibility* was confirmed by presenting the themes that could be anchored to participant quotations. The exemplar quotations are presented systematically for a visual *resonance* of the participants’ perceptions of using the height-adjustable sit-stand desks. Concerning s*ignificant contribution*, the authors describe the conceptual relevance of interrupt sitting time during office hours and the importance of informing further studies of environmental tools to reduce sedentary behaviour in the South African workplace. *Ethical* approval was obtained as described. Finally, *meaningful coherence* for this study was realised by ensuring robust methodology consistent with previous research of sedentary behaviour interventions in the workplace. ^[14]^

### Data analysis

Recordings were transcribed and de-identified by a professional service. All transcripts were read at least twice by each researcher and then coded line by line using a thematic analysis approach with Atlas.ti 9 (9.1.5.0, Atlas.ti Scientific Software Development GmbH). Two researchers (MP and PJG) read and coded the imported textual data to identify emergent themes. Discrepancies were discussed, and revisions were made until full consensus was achieved.

## Results

The participants (n=11) were mostly female (91%) and had a mean age 40.5 ± 12.6 years (Table 1). The majority had tertiary qualifications (91%) and 82% (n=9) were paid a monthly salary ≥R20000. The estimated self-reported sitting time ranged from 8.5 to 13.5 hours per day, with occupation-related sitting time contributing 76% to overall sitting time. Table 2 presents the themes with illustrative quotes.

### Overall impressions

#### Impact on ability to work

All participants commented on the workstations bearing on their ability to carry out vocational tasks. The participants reported that reading and responding to electronic mail was more comfortable in the standing position, while typing activities were best suited to the seated position.

#### Ease of use

Participants expressed enjoyment about using the workstation during the study period, specifically commenting on the improved work productivity and the innovative approach to office work. In support, some participants felt that the workstation helped reduce work-related boredom and fatigue.

### Enablers

#### Motivators

While most of the participants had never experienced using an adjustable sit-stand desk, many described seeing comparable products advertised and expressed a desire to experiment using it in their own personal work environment.

Participants agreed that sitting for extended periods during work hours resulted in musculoskeletal pain in the lumbar and cervical regions of the spine. They were therefore interested in using the workstation and a treatment modality to manage the occupation-related pain.

Discussions revolved around the potential application of the unit in the workplace and for their individual administrative duties. While most participants felt that they were encouraged to take part, some participants agreed it was an opportunity to reorganise their immediate work environment.

#### Perceived physical health benefits

Multiple participants recognised the health benefits prompted by the adjustable sit-stand desk. The interruption of sitting time was perceived as an evidence-based approach to supporting suitable seated and standing ergonomics, despite lengthy periods of being sedentary.

#### Perceived work benefits

For many of the participants, the adjustable sit-stand desk aided in improving their concentration during work responsibilities. Throughout the interviews, the participants discussed the improved job performance with using the desk, and few described the concept of skeletal muscle memory to adopt the routine of alternating sitting and standing.

#### Perceived behaviour modification

Participants talked about the influence of the adjustable sit-stand desks on their sitting and standing behaviour. They had various ideas about how the desks made them aware of the duration of sitting time and believed that they felt more active when they were in the standing position.

Some participants observed modifications in body position, such as a less slouched posture whilst in the standing position. Interestingly, the participants believed this position helped to reduce the chronic neck pain associated with typing for a long period of time while in a forward head posture, such as the sitting position at work.

### Obstacles/barriers

Despite having the adjustable sit-stand desk arranged using an individualised ergonomic procedure, some participants argued that the design restricted modification and believed that their own personal computers were not considered in the development of the unit.

Others explained, with emphasis, that although the height-adjustable sit-stand desks improved work focus, the units failed to accommodate their usual connected devices, such as printers that needed to be connected by cable.

#### Physical discomfort

For many of the participants, static standing for extended periods resulted in discomfort in the feet. Some participants saw that a change from standing to sitting relieved this discomfort.

One participant commented that the positional foot pain was managed by changing the standing position such as by shifting the body weight from one foot to the other.

### Use of the sit-stand workstation: sitting vs. standing

#### Features/novelties

For many of the participants, the notion of swapping between standing and sitting positions helped them to complete their tasks. For instance, participants perceived that they could complete tasks such as answering the telephone, responding to emails and conducting administrative duties in either sitting or standing positions. One participant felt that online meetings could be conducted in the standing position, while other participants believed that the sitting posture was best for answering the phone to respond to student queries.

#### Comfort

Although some participants described sitting to speak to staff and students on the telephone for five to ten minutes at a time, most of the participants reported being able to stand, intermittently for 15 to 30 minutes. Some participants were able to extend the time spent standing from 60 to approximately 90 minutes at a time, while others indicated that the mornings were better for reducing sitting time because they were more attentive and enthusiastic.

### Readiness to continue using height-adjustable sit-stand desks

All participants in the study indicated that they would like to continue using the height-adjustable sit-stand desks if they were made available to staff. Participants reported perceived improvements in work productivity and job satisfaction as the main reasons for supporting an initiative to reduce sedentary behaviour during work hours. Some participants reported that affordability was a limiting factor for not purchasing their own workstation.

## Discussion

The aim of this study was to describe how office workers in a South African university setting viewed the feasibility of environmental modifications, and in this study, sit-stand desks for reducing sitting time in the context of the work environment. Self-reported sitting time in the workplace was high yet aligns with previous data.^[6,7]^ Seven themes were developed including overall impressions of the height-adjustable sit-stand desks, motivation to experience the adjustable sit-stand desks, enablers of standing work using an adjustable sit-stand desk, use of sit-stand workstations, sitting versus standing, obstacles to using the adjustable sit-stand desk in the standing position, readiness to continue using sit-stand desks, and perceived behaviour modification. These themes are focused on healthy workplace behaviours and enhancements to occupational responsibilities, and they present an understanding of the way office workers perceive the promotion of interrupting sedentary time as essential.

In agreement with existing evidence ^[8]^, data in our study showed a general acceptance of height-adjustable sit-stand desks to interrupt sedentary time without disrupting usual work responsibilities such as email communication and meeting attendance. Other studies reported a different method to interrupt occupational sitting time, with participants using activities, such as walking between meetings, using the toilet, printer or getting coffee to break prolonged sitting. ^[15]^ Consistent with our findings, a study that investigated the lived experiences of office employees with prior use of sit-stand workstations, ^[16]^ illustrated that the substitution of a traditional work model (task completion in the seated position) with a sit-stand desk in the workplace can reduce vocational sitting time and improve productivity. Additionally, previous research has reported participants’ variation in the usage of the sit-stand desk stands, with four studies stating the use of the workstation had no influence and three indicated that it enhanced productivity. ^[17]^ Therefore, modifying the workspace by the alternative of between sitting and standing might be useful to reduce the monotonous feeling of fatigue and boredom.

Consistent with previous literature, our results indicated that the individual’s willingness to adopt the height-adjustable sit-stand desks in their work environments were mostly precipitated by personal and organisational motives. ^[18]^ In addition, our findings highlighted that the personal and organisational influences for participation in this study were driven by curiosity to experiment with the compatibility of the workstation and other anticipated health benefits. Participants with existing musculoskeletal conditions were motivated to participate in the study in order to attain perceived health benefits through the alternative between sitting and standing transitions. As noted in previous studies, our results show that participants spend most of their time at work seated, which makes them prone to adverse musculoskeletal conditions ^[19]^ Alternating between sitting and standing positions was perceived as less comforting for work activity by some participants ^[20]^ however, the study by Karakolis et al. ^[17]^ showed that working in the standing position was associated with chronic lumbar pain reduction in some employees. The acceptance of sit-stands desks in the work environment should nevertheless be investigated further as there might be resistance to change despite demonstrable improvement in physical health.

Consistent with previous research, ^[21, 22]^ participants in our study were also motivated to use the sit-stand desk because of the perceived benefits to musculoskeletal health, such as the reduction in chronic lower back pain due to the extended sitting time in the various domains of sedentary behaviour. Occupation-related sitting contributed the most to overall daily sitting, a finding that has been observed in a number of systematic reviews investigating the sedentary behaviours of office workers. ^[8, 23]^ Participants were also encouraged to use the workstations because of the incidental ergonomic intervention provided by the researchers during the initial setup of the workstations and continued engagement throughout the study. Other studies have shown acute and chronic improvements as a result of the correction of working postures, ^[24, 25]^ but this needs to explored further in this study population.

From the perspective of employee health, the findings of our study demonstrate that there were many occupation-related benefits that may be achieved through workplace interventions. Specifically, some of the advantages described by participants included improvements in work productivity and mental concentration. Indeed, in one study, improvements in task engagement in the erect position, despite there being reported discomfort. ^[20]^ Evidence is inconsistent regarding productivity, one demonstrating that job performance was not hindered by standing work. ^[26]^ The present study’s findings observed that participants viewed the standing position as better for overall job performance compared to the seated position. These data illustrate that in the context of the office environment, tasks conducted in the standing position could also have varying influence on deliverables, depending on the task characteristics, duration allocated for task completion, and the expectations of line management. Participants in the present study described improvements in attentiveness in the standing position compared to sitting for the completion of tasks, a finding which is in agreement with contemporary evidence. ^[27, 28]^

### Limitations

This study has limitations worth noting, including the small sample size and the lack of information explaining why people declined to volunteer to participate in the study. Participants in this study were university office staff, primarily women, with only one male who participated in the study, and may therefore not be representative of other office workers. In addition, breaks in sitting time were self-reported and no objective measures of free-living data were collected. The transport domain is important and travel to and from work could affect postural choice during the participant's work hours; however, understanding the nature of this domain is outside the scope of this feasibility study and should be examined in future research. This study was conducted during the stricter COVID-19 lockdown measures in South Africa, which limited face-to-face social interaction with the participants. However, the researchers continued engagement with participants using online and telephonic communication. This sample consisted of participants that presumably earn a monthly salary that is higher than other South Africans and may therefore not be applicable to low-income workers in LMICs. Finally, study participants provided information about musculoskeletal injuries that should be considered in future research studies.

## Conclusion

The participants in this study described their experiences using the height-adjustable sit-stand desk. The overall sentiment was that the workstation would be accepted in the workplace given the potential for sitting less, perceived improvements in productivity, and enhanced physical and mental health. The findings of this study suggest that there is a need for the modification of the occupational environment to reduce sitting time.

## Supplementary Information



## Figures and Tables

**Table 1:** Demographic characteristics (n=11)

Characteristic	N (%)
Age (years)*	40.5 ± 12.6
Female	10 (90.9)
**Highest level of education**	
Completed high school	1 (9.1)
Diploma/ College certificate	1 (9.1)
University degree	3 (27.3)
Postgraduate degree	6 (54.4)
**Monthly income**	
Prefer not to answer	1 (9)
<R15000	1 (9)
R15000–R19999	0 (0)
R20000–R24999	4 (36)
R25000–R29999	2 (18)
≥R30000	3 (27)
**Estimated sedentary behaviour** *	
Occupational sitting (hours/day)	8 (6.0–8.0)
Sitting during commuting (hours/day)	1 (0.5–1.5)
Sitting at home (hours/day)	2 (2.0–5.0)
Total sitting time (hours/day)	10.5 (8.5–13.5)

* Data are expressed as mean ± SD or median (interquartile range).

**Table 2:** Themes with illustrative quotes

Theme	Illustrative quotes
**Overall impressions**	It was a positive one, eh, user friendly, you know, you’d like, it was useful, it was easy to use, easy to move around with the thing. To move the screens, it was not hard work, if I am making any sense at all. *(Participant 1B89, female, aged 47 years)*
**Enablers**	I always, always try and keep like, if it's an invisible upright posture, I was trying to keep an upright posture even when I'm driving but my like, you know, rear mirror you know, being tilted a little bit higher up to force you to sit upwards and when I'm sitting at my table that kind of stuff ah, I'll be typing something and that kind of stuff in them in that moment represent we read over to make sure everything is fine, then I'll sit up straight like that. *(Participant A11, male, aged 24 years)*
**Obstacles/barriers**	I would have liked it if it had a one grade lower for the position of the laptop. So, I know that its design….it is not designed for laptop it is designed for a monitor but even at my eye level I did find myself having to adjust quite a bit, because it was slightly higher than what I was used to. Not slightly higher, it was slightly higher than what eye level would be, but like I am talking centimetres here, because usually, you know, I am quite used to looking down at something. So that gradient from normally looking down and then all of a sudden eye level and slightly higher than eye level, it is a bit of an adjustment. So if it had, you know those that where you can adjust the levels of the platform, if it had it one lower that would have been perfect, from that bottom rung. *(Participant 1D53, female, aged 37 years)*
**Use of sit-stand workstation sitting vs. standing**	I used little shelves and like you said that novel hook was very nice, my phone I never needed to look for it because it’s always under paper. *(Participant 1B12, female aged 66 years)* I stand a lot during the day so when you get a chance to sit down you take it *(Participant 87C, female, aged 24 years)*
**Readiness to continue using height-adjustable sit-stand desks**	Look if money was no object, so if money was no object, and these things were for free, yes, I would definitely. And I'd probably I mean, if you could walk me into a factory or a store that had all these ergonomic stuff in and I could just take off the shelf and test it and put it on my desk, then probably what I would, I would set up my home station as well. My home station right now as a dining room table. And a dining room chair where the cushioning has gone, so I would set up, if money was no object, I'd set up home to be able to be flexible and move around. And then work, office work, I would definitely also set up permanently to be able to move around and do stuff. *(Participant 1E64, female, aged 52 years)*
