# Supplementary material for: “If money was no object”: A qualitative study of South African university office workers’ perceptions of using height-adjustable sit-stand desks
Source: S Afr J Sports Med. 2022 Jan 1;34(1):v34i1a13881. doi: 10.17159/2078-516X/2022/v34i1a13881 (PMC9924588; doi:10.17159/2078-516X/2022/v34i1a13881)
Supplement: Supplementary file 1 [file 2078-516X-34-v34i1a13881-s001.pdf]

"If money was no object": A qualitative study of South African university office workers' perceptions of using height-adjustable sit-stand desks

Supplemental Digital Content 1: Interview guide

1. What motivated you to participate in the trial of the sit-stand workstation?
2. What were your general impressions of using the sit-stand workstation?

Prompts

a. *How much did you use the sit-stand workstation?*
b. *Was there anything about using the sit-stand workstation that you particularly liked?*
c. *Was there anything that you particularly disliked?*
d. *Any comments about the work surface attached to the workstation?*

3. What types of tasks did you generally do standing up?

Prompts

a. *Where there times of the day when you stood more?*
b. *What made you change from standing to sitting?*
c. *Were there any reasons for those decisions?*

4. What types of tasks did you generally do sitting down?

Prompts

a. *Where there times of the day when you sat down more?*
b. *What made you change from sitting to standing?*
c. *Were there any reasons for those decisions?*

5. Did anything encourage you to stand up more to complete your work?

Prompts

a. *Were other people around you using a workstation?*
b. *Did certain types of footwear make it easier?*
c. *Were you able to stand for long periods over time?*
d. *How long did you tend to stand up for each time?*

6. Was there anything that stopped you from standing more than you did?

Prompts

a. *Did being in an open plan office make any difference?*
b. *Were there any tasks that were not practical while standing?*
c. *Was it comfortable to stand and work?*
d. *Did you have any injuries or other personal factors?*

7. What types of physical changes did you notice from using the workstation?

Prompts

a. *Any changes in posture?*
b. *Any musculoskeletal changes?*
c. *Any changes in tiredness or energy levels?*
d. *Were these related to using the workstation?*

8. What types of changes in your work performance did you notice from using the sit-stand workstation?

Prompts

a. *Any effect on productivity?*
b. *Any effect on the ability to concentrate?*

9. Would you continue to use the sit-stand workstation if you could?

Prompt: *why/why not?*

In closing, is there anything else you'd like to say about your experience of using the workstation or about your experience of wearing the activity monitors?
